# Potential of gut-derived short-chain fatty acids to control enteric pathogens

**DOI:** 10.3389/fmicb.2022.976406

**Published:** 2022-09-20

**Authors:** Ziyang Zhan, Hao Tang, Ying Zhang, Xinxiang Huang, Min Xu

**Affiliations:** ^1^Department of Gastroenterology, Affiliated Hospital of Jiangsu University, Zhenjiang, Jiangsu, China; ^2^Department of Biochemistry and Molecular Biology, School of Medicine, Jiangsu University, Zhenjiang, Jiangsu, China; ^3^Institute of Digestive Diseases, Jiangsu University, Zhenjiang, Jiangsu, China

**Keywords:** short-chain fatty acids, enteric pathogens, post-translational modifications, acetate, propionate, butyrate

## Abstract

Short-chain fatty acids (SCFAs) are a very important group of metabolites located in the gut that play a crucial role in the regulation of gut function and pathogen resistance. Since many enteric pathogens respond differently to various SCFAs, substantial efforts have been made to understand the regulatory effects of SCFA types on enteric pathogens. The application of protein post-translational modifications (PTMs) in bacterial research provides a new perspective for studying the regulation of enteric pathogens by different SCFAs. Existing evidence suggests that the SCFAs acetate, propionate, and butyrate influence bacterial processes by extensively promoting the acylation of key bacterial proteins. SCFAs can also prevent the invasion of pathogenic bacteria by regulating the barrier function and immune status of the host gut. In this review, we describe the mechanisms by which different SCFAs modulate the pathogenicity of enteric pathogens from multiple perspectives. We also explore some recent findings on how enteric pathogens counteract SCFA inhibition. Lastly, we discuss the prospects and limitations of applying SCFAs to control enteric pathogens.

## Introduction

Intestinal health is closely associated with overall human health. A healthy gut harbors a diverse community of commensal bacteria commonly referred to as the microbiota (Martens et al., 2018). An important function of the gut microbiota is digestion of dietary fiber. Bacterial fermentation of dietary fiber produces short-chain fatty acids (SCFAs) that influence host physiology and gut immune responses and have a profound impact on the survival and virulence of enteric pathogens (Buffie and Pamer, 2013; Zhang et al., 2020).

The occurrence of intestinal disease is thought to be related to diet and lifestyle, including reduced consumption of fiber polysaccharides. The most prevalent enteric pathogens include *Shigella* spp., *Escherichia coli*, *Clostridium difficile*, and *Salmonella* spp., which can cause a range of intestinal diseases and complications by disrupting intestinal homeostasis (Rivera-Chavez and Baumler, 2015; Abt et al., 2016). Antibiotic therapy often destroys the natural gut microbiota, promoting the infection and proliferation of antibiotic-resistant enteric pathogens. Controlling the commensal microbiota to modulate metabolite production can be used to prevent or even cure infections caused by pathogenic bacteria (Ricke, 2003; Mekonnen et al., 2020).

Short-chain fatty acids are end-products of the fermentation of undigested dietary carbohydrates, mainly dietary fiber, by the gut microbiota. It is generally believed that the effects of SCFAs on host health are beneficial (Serino, 2019). In addition to their important roles in host metabolism and immunity, SCFAs are also vital in intestinal pathogen resistance and the maintenance of intestinal microbiota homeostasis (Rooks and Garrett, 2016; Blander et al., 2017; Pickard et al., 2017). SCFAs, mainly acetate, propionate, and butyrate, have been shown to play diverse roles in the regulation of pathogenic bacteria. With the application of protein modification omics, in addition to studying the regulatory relationship between SCFAs and pathogenic bacteria, determining the regulatory effects of each component on bacterial metabolism and virulence has attracted much attention. Recent studies suggest that SCFAs hold great potential, since the metabolic intermediates of different SCFAs are widely involved in the post-translational modifications (PTMs) of important bacterial proteins and regulate the pathogenicity of enteric pathogens. Therefore, a comprehensive understanding of the link between SCFAs and enteric pathogens is essential.

In this review, we summarize current knowledge about the effects of three major SCFAs on enteric bacteria, including the catabolism of SCFAs in bacteria, advances in SCFA regulation of bacterial virulence, and novel insights into regulatory mechanisms. In addition, we discuss the indirect regulatory effects of SCFAs on enteric bacteria, such as acting as nutrients, maintaining the gut barrier, and modulating host immunity. Although we primarily focus on regulation of the enteric bacteria, examples from other microorganisms are included and parallels are drawn when possible. Moreover, methods used by pathogenic bacteria to counteract SCFAs are also discussed. Therefore, caution must be taken when considering the use of SCFAs for the control of infections caused by enteric pathogens.

## Production and metabolism of short-chain fatty acids in the gut

Short-chain fatty acids are the main metabolites of anaerobic fermentation of non-digested carbohydrates in the gut (Morrison and Preston, 2016). The diversity of the gut microbiota and the residence time of food in the gut play an important role in the production of SCFAs (Macfarlane and Macfarlane, 2003; Canfora et al., 2015). The most abundant SCFAs in the gut include acetate, propionate, and butyrate, which together account for approximately 95% of all SCFAs (Rios-Covian et al., 2016). The conversion of dietary fiber to SCFAs in the gut is mediated by the specific members of the gut microbiota, and the known pathways are shown in Table 1. Acetate, the most abundant SCFA, can be produced from pyruvate *via* acetyl-coenzyme A (acetyl-CoA) and the Wood–Ljungdahl pathway (Ragsdale and Pierce, 2008). Acetate is produced by a diverse range of human colonic microbiota, such as *Akkermansia muciniphila*, *Bacteroides* spp., and *Bifidobacterium* spp. (Hetzel et al., 2003; Rey et al., 2010; Louis et al., 2014). Another major SCFA, propionate, is mainly produced from succinate by *Bacteroides* spp., *Phascolarctobacterium succinatutens*, *Dialister* spp., and *Veillonella* spp. *via* the succinate pathway. Propionate can also be synthesized from acrylate with lactate as a precursor through the acrylate pathway by *Megasphaera elsdenii* and *Coprococcus catus* (Scott et al., 2006), and the propanediol pathway by *Salmonella* spp., *Roseburia inulinivorans*, and *Ruminococcus obeum* (Duncan et al., 2002). The third major SCFA, butyrate, is generally formed by condensation of two molecules of acetyl-CoA *via* either butyrate kinase or the butyryl-CoA: acetate CoA-transferase route by bacteria such as *Coprococcus comes* and *Coprococcus eutactus*. Butyrate can also be produced through an alternative route by the use of exogenous acetate (Louis et al., 2004). Furthermore, there are also microbial pathways for butyrate formation from carbohydrates, organic acids, glutamate, and lysine in the gut community (Louis and Flint, 2017).

**TABLE 1:** Pathways and bacterial groups contributing to short-chain fatty acid (SCFA) formation.

SCFAs	Pathway/route	Producer	References
Acetate	Acetyl-CoA route	*Akkermansia muciniphila*, *Bacteroides* spp., *Bifidobacterium* spp., *Prevotella* spp., *Ruminococcus* spp.	Ragsdale and Pierce, 2008; Rey et al., 2010; Louis et al., 2014
	Wood–Ljungdahl pathway	*Blautia hydrogenotrophica*, *Clostridium* spp., *Streptococcus* spp.	
Propionate	Succinate pathway	*Bacteroides* spp., *Dialister* spp., *Phascolarctobacterium succinatutens*, *Dialister* spp., *Veillonella* spp.	Duncan et al., 2002; Scott et al., 2006; Koh et al., 2016
	Acrylate pathway	*Coprococcus catus*, *Eubacterium hallii*, *Megasphaera elsdenii*, *Veillonella* spp.	
	Propanediol pathway	*Roseburia inulinivorans*, *Ruminococcus obeum*, *Salmonella enterica*	
Butyrate	Classical pathway *via* butyrate kinase	*Coprococcus comes*, *Coprococcus eutactus*	Louis et al., 2004; Louis and Flint, 2017
	Butyryl-CoA:acetate CoA-transferase route	*Anaerostipes* spp., *Coprococcus catus*, *Eubacteriumrectale*, *Eubacterium hallii*, *Faecalibacterium prausnitzii*, *Roseburia* spp.	
	Organic acids, glutamate, and lysine route	*Acidaminococcus fermentans*, *Clostridium sporosphaeroides*, *Clostridium symbiosum*, *Fusobacterium* spp., *Peptostreptococcus asaccharolyticus*	

**FIGURE 1:**
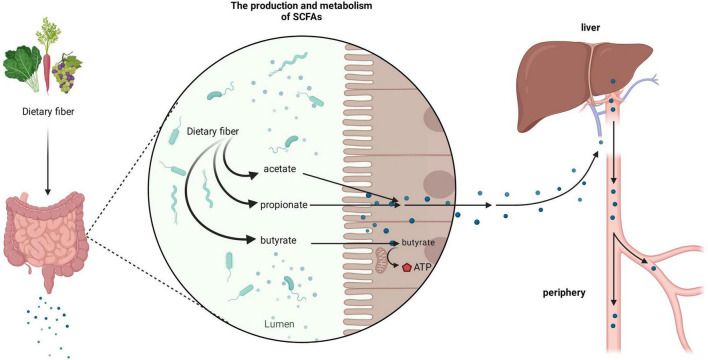
Production and metabolism of short-chain fatty acids (SCFAs). After dietary fiber ingested by the human body reaches the gut, it can be broken down and utilized by the rich microbiota to produce various forms of SCFAs, such as acetate, propionate, and butyrate. Among them, butyrate is mainly used by colon cells as an energy substance, whilst acetate and propionate enter organs such as the liver through the portal vein and enter the periphery.

It is important to consider the biological gradient of SCFAs across various tissues to fully understand their biological effects in humans. The concentration of SCFAs varies in different parts of the intestine; the concentration in the cecum and proximal colon is the highest, whereas it decreases in the distal colon (Koh et al., 2016). After SCFAs are produced in the intestine, most of them can be taken up by epithelial cells, through the portal vein into the liver, or into systemic circulation. Only a minor portion can be excreted in feces (Cummings et al., 1987; Topping and Clifton, 2001; den Besten et al., 2013). Among them, especially, butyrate can be rapidly metabolized by colonocytes as the preferred energy source while the remaining SCFAs are transported into the portal vein to functional organs and tissues. Propionate is mainly metabolized in the liver, while acetate enters the periphery and becomes the most abundant SCFA in peripheral circulation (Figure 1; Cummings et al., 1987; Bloemen et al., 2009).

## Direct regulation of enteric pathogens by short-chain fatty acids

Different parts of the gut with various compositions and concentrations of SCFAs show great differences in the regulation of bacterial activities and even opposing regulation patterns. Studies have shown that different concentrations of SCFAs have distinct regulatory effects on bacterial virulence, and SCFAs in the colon show more obvious inhibitory effects than those in the ileum. The ileal SCFAs, with acetate being the most abundant, induce expression of the *Salmonella* pathogenicity island-1 (SPI-1) gene, which promotes the ability of bacteria to invade epithelial cells (Lawhon et al., 2002). However, the colonic SCFAs have a significant inhibitory effect on *Salmonella* invasion due to the increased proportion of propionate and butyrate (Gantois et al., 2006; Hung et al., 2013; Hockenberry et al., 2021). Intestinal microbes mediate colonization resistance in various ways. Microbe-derived SCFAs are a group of symbiotic metabolites that can mediate microbial colonization resistance to enteric pathogens. Several current studies have demonstrated the importance of SCFAs in the regulation of enteric pathogens. However, little is known about the specific mechanisms by which SCFAs affect enteric pathogens. In view of the varying effects of different components and concentrations of SCFAs, in addition to focusing on SCFAs as a whole, the interactions of diverse SCFAs with enteric pathogens have been investigated, and the exact mechanisms involved are gradually being elucidated (Table 2).

**TABLE 2:** The mechanism of short-chain fatty acids (SCFAs) in the regulation of pathogenic bacteria.

SCFA type	Pathogens	Effect of SCFAs	Mechanism	References
Acetate	*S. typhimurium*	Promote bacterial invasion ability	Phosphorylation of response regulators SirA	Lawhon et al., 2002
	EPEC	Promote bacterial adhesion and motility	Activate master regulator of LEE genes	Yang et al., 2018
	*S. typhimurium*	Attenuate the virulence	Acetylation of key bacterial proteins	Ren et al., 2017, 2019
	*S. typhimurium*	Activate SPI-1 gene expression	Involvement in transcriptional crosstalk control of the flagellar system as a nutrient	Hamed et al., 2019
Propionate	*S. typhimurium*	Inhibit bacterial growth	Toxic by-products; disrupt intracellular pH homeostasis	Horswill et al., 2001; Ricke, 2003; Jacobson et al., 2018
	MRSA	Inhibit bacterial growth	Interfere with bacterial metabolism	Jeong et al., 2019
	AIEC	Promote bacterial adhesion and biofilm formation	Induce an increased level of proteins, which are known pathogenicity factors	Ormsby et al., 2020; Pobeguts et al., 2020
	*S. typhimurium*	Reduce bacterial invasion ability	Promote the post-translational modification	Hung et al., 2013
	*L. monocytogenes*	Inhibit bacterial growth; alter carbon metabolism	Affect the production of the critical virulence factor listeriolysin O	Rinehart et al., 2018
Butyrate	*S. typhimurium*	Attenuate the virulence	Affect bacterial metabolism	Bronner et al., 2018
	EHEC	Promote bacterial adhesion	Activate a leucine-responsive regulatory protein Lrp	Nakanishi et al., 2009
	*S. typhimurium*	Reduce the invasion ability	Promote the post-translational modification	Gantois et al., 2006; Zhang et al., 2020
	*L. monocytogenes*	Inhibit virulence factor production; compromise bacterial resistance to antibiotic	Compromise listeriolysin O production	Sun et al., 2012

### Mechanisms of acetate regulation

Acetate accounts for the highest proportion of SCFAs in the gut. Direct roles of acetate in modulating the virulence of pathogens are well-reported. For example, previous research has shown that acetate can act as an environmental signal to promote the expression of *Salmonella* invasion genes. It has also been shown that the acetyl phosphate (AcP) formed by the metabolism of acetate in bacteria causes the BarA/SirA two-component system to be phosphorylated, leading to the regulation of SPI-1 gene expression, which is also dependent on low pH (Lawhon et al., 2002). Acetate can also synergize with yeast extract to activate SPI-1 gene expression in *Salmonella*, promoting the invasive ability of bacteria (Hamed et al., 2019). In addition to the effect of acetate on the virulence of *Salmonella*, another study found that acetate activated the expression of the locus of enterocyte effacement (LEE) and flagella genes in enteropathogenic *E. coli* (EPEC) grown at different pH values, thereby promoting the adhesion and motility of EPEC (Yang et al., 2018). In EPEC, genetic determinants involved in the development of attaching and effacin lesions are primarily located on pathogenicity islands referred to as LEEs (Garmendia et al., 2005).

**FIGURE 2:**
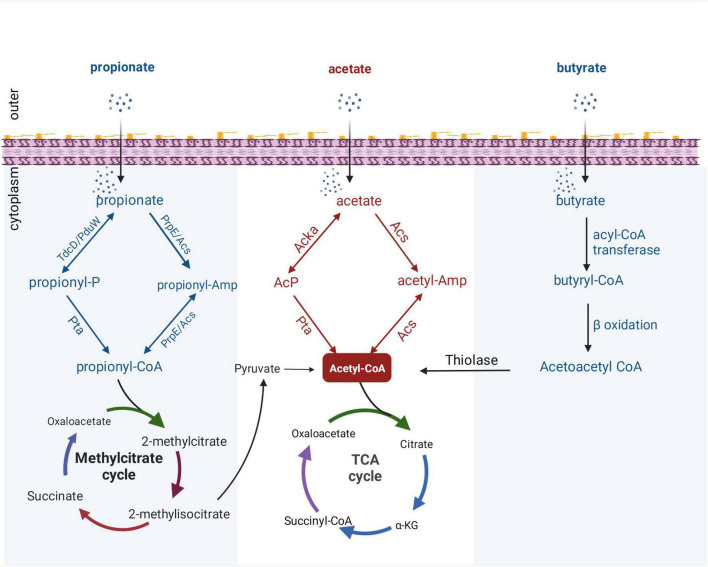
Metabolic pathways of SCFAs in bacteria. All catabolic pathways for acetate, propionate, and butyrate require these SCFAs to be activated to their corresponding acyl-CoA forms before being converted to metabolites that can enter central metabolism. Acetyl-CoA can enter the tricarboxylic acid (TCA) cycle directly, while propionyl-CoA can be catabolized through a number of different pathways, converted to pyruvate, acetate, or succinyl-CoA, and then enter the TCA cycle. Butyryl-CoA is catabolized by β-oxidation, and thiolase generates acetyl-CoA and then enters the TCA cycle. PduW/TdcD, propionate kinase; Pta, phosphotransacetylase; PrpE, propionyl-CoA synthase; Acs, acetate kinase; propionyl-P, propionyl-phosphate.

In addition to the mechanisms described above, which regulate enteric pathogens by regulating the transcription of genes, acetate may directly regulate the activity of virulence factors through PTMs of proteins. In recent years, protein modification omics research has become increasingly extensive, and many review articles have described the regulatory role of acetylation modification in bacterial physiology and metabolism (Ren et al., 2017; Liu et al., 2021). Acetyl-CoA and AcP are the central metabolites of SCFAs in bacterial metabolism and act as acyl donors to promote the acetylation of bacterial proteins. In bacteria, acetate is metabolized to acetyl-CoA in two ways (Figure 2): one is the synthesis of acetyl-CoA from acetate by acetyl-CoA synthase (Starai et al., 2002), and the other is through acetate kinase and phosphotransacetylase, which reversibly converts acetate to acetyl-CoA *via* high-energy AcP intermediates (Weinert et al., 2013). PTMs in bacteria are diverse, and they can affect protein structure and function. Previous work has shown that supplying acetate exogenously increases the intracellular concentration of AcP and promotes phosphorylation of protein BarA, thus activating invasion gene expression in *Salmonella* (Lawhon et al., 2002). However, the regulatory roles of acetylation in bacterial physiology and metabolism may differ from phosphorylation. Some studies have found that adding acetate to growth medium can increase the level of protein acetylation in *Salmonella* and *E. coli* (Schilling et al., 2015; Wolfe, 2016; Ren et al., 2019). In *Salmonella*, the protein PhoP, which is closely related to virulence, can be acetylated, and the acetylation inhibits PhoP’s DNA-binding ability, thus impairing *Salmonella* virulence (Ren et al., 2019). In addition, several studies have confirmed that acetylation modification can regulate acid resistance (Ren et al., 2015) and virulence-related proteins in *Salmonella* (Qin et al., 2016; Sang et al., 2016).

It seems that the regulation of phosphorylation and acetylation by acetate metabolism produces contradictory results, but these contradictions may be explained by the cross-talk of PTMs. Acetylation of K102 of PhoP in *Salmonella typhimurium* (*S. typhimurium*) has been shown to inhibit PhoP activity (Ren et al., 2019). This PTM crosstalk phenomenon is widespread in eukaryotes and prokaryotes (Barak and Eisenbach, 2004; Dai and Gu, 2010); many such phenomena are yet to be discovered and described. Therefore, additional proteomic studies are needed to better elucidate the complex functions of acetate in regulating pathogen pathogenesis.

### Mechanisms of propionate regulation

Propionate is another major SCFA in the gut and is produced by anaerobic bacterial fermentation. It is widely used in the food industry to protect baked goods from microbial contamination because of its microbial inhibitory properties (Wales et al., 2010).

The direct regulatory effect of propionate on enteric pathogens is mainly reflected in growth inhibition. Early studies have found that toxic metabolites produced during propionate catabolism can inhibit the growth of *S. typhimurium*, and propionyl-CoA plays a key role in this process. However, the specific mechanism of antibacterial activity is largely unclear (Horswill et al., 2001). Later studies found that SCFAs can diffuse across bacterial cell membranes and dissociate into anions and protons after entering the cytoplasm, thereby inhibiting bacterial growth (Ricke, 2003). Furthermore, previous studies have shown *Bacteroides* spp. protect mice from *S. typhimurium* infection by increasing intestinal propionate levels, and it has been shown that propionate directly inhibits pathogen growth *in vitro* by disrupting intracellular pH homeostasis (Jacobson et al., 2018). In addition, some researchers have found that propionate can effectively inhibit the growth of methicillin-resistant *Staphylococcus aureus* (MRSA) both *in vitro* and *in vivo*, effectively reducing MRSA infection (Jeong et al., 2019).

While propionate has a broad inhibitory effect on the growth of many bacteria, it has also been found that exposure of adherent-invasive *E. coli* (AIEC) to propionate stimulates adhesion and biofilm formation, inducing a range of toxicities (Ormsby et al., 2020). Similarly, propionate was reported in another study to induce the expression of some important pathogenic factors in AIEC, including porins, transcription factors, and proteins involved in protection against oxidative stress and cell wall biogenesis (Pobeguts et al., 2020). Although propionate stimulation promotes AIEC virulence, it also induces an inflammatory response in the gut, preventing the increase in AIEC adhesion and invasion (Pace et al., 2021), which reflects the complexity of the mechanism by which SCFAs regulate bacterial virulence.

With the increased attention on the application and development of protein modification omics, propionate has been shown to induce the overall propionylation of eukaryotic and prokaryotic proteins (Sun et al., 2016; Hogh et al., 2020). Propionyl-CoA, an intermediate metabolite of propionate, plays an important role in propionylation (Sun et al., 2016). Generally, after propionate is taken up by gut bacteria, propionyl-CoA can be synthesized from propionate through two distinct pathways: reversible conversion of propionate to propionyl-CoA by propionate kinase and phosphotransacetylase; and *via* propionyl-CoA synthase or alternative acetyl-CoA synthase (Palacios et al., 2003; Liu et al., 2014). After the formation of propionyl-CoA, it can enter the methylcitrate cycle for degradation, and finally produce acetyl-CoA which enters the tricarboxylic acid (TCA) cycle (Figure 2). Although progress has been made on bacterial propionyl-CoA synthesis pathways, little is known about regulation of bacterial processes by propionylation of bacterial proteins. Hung et al. (2013) found that propionate can regulate the expression of *Salmonella* invasion-related genes by promoting the PTM of the invasion transcriptional regulator HilD and reduce the invasion ability of *S. typhimurium* on HEp-2 cells. In addition, a proteomic study revealed a wide range of propionylation substrates in *E. coli* and suggested new roles of lysine propionylation in bacterial physiology (Sun et al., 2016). Propionate-promoted PTMs are similar to acetylation modifications, which broadly regulate bacterial growth and virulence, but the specific regulatory mechanisms remain largely unknown. Thus, elucidating the mechanistic understanding of how intestinal propionate affects the gene regulation and pathogenesis of pathogenic bacteria still requires further research.

### Mechanisms of butyrate regulation

The effect of butyrate on virulence gene expression *in vitro* has been tested during *Salmonella* interactions with the host using both tissue culture and animal infection models. Early studies have found that butyrate can specifically downregulate the expression of the SPI-1 gene and inhibit the invasion ability of *S. typhimurium*, but the molecular mechanism by which butyrate inhibits the expression of the SPI-1 virulence gene was not clear at the time (Lawhon et al., 2002; Gantois et al., 2006). Later, some researchers indirectly found an inhibitory effect of butyrate on intestinal pathogenic bacteria in animal experiments. When the abundance of symbiotic *Clostridium* butyrate-producing bacteria in the intestinal cavity of mice treated with streptomycin was significantly reduced, it led to the expansion of *S. typhimurium* in the gut (Rivera-Chavez et al., 2016). However, the mechanism by which butyrate limits pathogen expansion is not fully resolved. Since butyrate was shown to be the main energy source for colonocytes, the inhibitory effect of butyrate on pathogens may be due to the significant effects on host cell physiology (Donohoe et al., 2012). Interestingly, butyrate can also participate in bacterial metabolic regulation as a carbon source and directly attenuate the virulence of enteric pathogens by affecting bacterial metabolism (Bronner et al., 2018). In addition to the inhibition of the virulence of enteric pathogens by butyrate mentioned above, it has been proposed that butyrate, at a certain concentration range, enhanced the expression of virulence genes related to EHEC colonization in the gut; therefore, the amount of butyrate in the gut may affect EHEC infectivity, LEE gene expression, and the ability of the organism to adhere to epithelial cells (Nakanishi et al., 2009). Overall, these findings reflect the complexity of butyrate’s regulation on bacterial virulence.

It was mentioned above that butyrate can modulate virulence by affecting bacterial metabolism. To the best of our knowledge, the metabolic processing of butyrate after ingestion by bacteria has not been fully explained. Under aerobic conditions, E. coli can use fatty acids as sole carbon and energy sources. However, long-chain fatty acids of at least 12 carbon atoms are required for the induction of the catabolic enzymes. Campbell et al. (2003) revealed a new aerobic β-oxidation pathway for fatty acid degradation in *E. coli*; YdiD proteins can support anaerobic fatty acids utilization in the presence of nitrate. Interestingly, it was found that butyrate can be utilized by anaerobic oxidation in *S. typhimurium* that expresses the ydiQRSTD operon, but the activation of this pathway is strictly anaerobic and occurs in the presence of electron acceptors (Bronner et al., 2018). Briefly, after uptake through the cell membrane, butyrate is converted into butyryl-CoA by acyl-CoA transferase, and butyryl-CoA can form acetoacetyl-CoA through β-oxidation. Finally, acetyl-CoA is synthesized by thiolase and enters the TCA cycle (Figure 2).

In recent years, protein acylation has been proven to be ubiquitous in bacteria (Hentchel and Escalante-Semerena, 2015). Butyryl-CoA produced from butyrate catabolism can serve as a substrate for acyltransferase to catalyze lysine butyrylation (Chen et al., 2007). Through the analysis of PTMs, the mechanism by which butyrate regulates bacterial virulence has become more and more clear. Studies have shown that butyrate can acylate the transcriptional regulator HilA of *Salmonella* SPI-1 at several key lysine residues, resulting in the downregulation of SPI-1 genes, further enriching the mechanism by which butyrate regulates bacterial virulence (Zhang et al., 2020), confirming the potential of butyrate as a metabolite to modulate the virulence of pathogenic bacteria. Collectively, these studies suggest that the mechanism of butyrate in regulating enteric pathogen virulence is likely due to the PTMs of many important bacterial functional proteins; however, more research is required to identify these targets.

## Indirect regulation of enteric pathogens by short-chain fatty acids

In addition to directly affecting bacterial growth and virulence, SCFAs can protect against colonization and infection of enteric pathogens by maintaining the epithelial barrier and associated immune tissue functions (Peng et al., 2009; Jung et al., 2015). As important nutrients in the gut, SCFAs can maintain the nutritional environment of the intestine, promote the growth of commensal bacteria, and stabilize the intestinal epithelial barrier. Activation of immune mechanisms can result in the direct killing of microbes or local or systemic tolerance of pathogens.

### Nutrient and barrier protection of short-chain fatty acids

The first line of host defense against invasion by enteric pathogens is the intestinal mucosal barrier (Turner, 2009). In a healthy gut, with SCFA concentrations as high as 130 mM in the proximal colon, butyrate acts as the preferred energy source for intestinal epithelial cells to maintain energy homeostasis (Hamer et al., 2008; Donohoe et al., 2011), and propionate can enter the TCA cycle *via* succinyl-CoA as an energy source (Waldecker et al., 2008). Acetate is also an essential SCFA that regulates the homeostatic metabolic state (Erny et al., 2021).

Short-chain fatty acids can maintain an anaerobic environment in the gut in several ways. For example, butyrate polarizes intracellular metabolism to mitochondrial β-oxidation of fatty acids by activating PPAR-γ signaling, increasing oxygen consumption in terminally differentiated colonocytes to maintain an anaerobic environment in the gut (Alex et al., 2013; Litvak et al., 2018). The anaerobic environment of the gut is conducive to the growth of commensal anaerobic bacteria in the gut microbiota whilst controlling the invasion and expansion of enteric pathogens (Faber et al., 2016; Litvak et al., 2017). Furthermore, SCFAs play important roles in enhancing the epithelial barrier in various ways. Studies have found that SCFAs can regulate the secretion of antimicrobial peptides from the intestinal epithelium to enhance epithelial barrier function (Zhao et al., 2018). SCFAs also appear to play an important role in regulating the integrity of the epithelial barrier through coordinated regulation of tight junction proteins (Cani et al., 2008; Wang et al., 2012).

### Regulation of the immune system

The intestine is a unique immunological site where host-microbiota interaction occurs, and enteric pathogens often lead to infectious diseases by disturbing the equilibrium between the host immune system and microbiota. Substantial evidence suggests that microbiota-derived SCFAs, including acetate, propionate, and butyrate, play critical roles in maintaining immune homeostasis through multiple mechanisms. In fact, SCFAs have a global anti-inflammatory effect mainly by up-regulating anti-inflammatory and down-regulating pro-inflammatory cytokines, which consequently promote immune homeostasis, resulting in a protective response against pathogens (Maslowski et al., 2009).

Short-chain fatty acids can be sensed by the immune system; G protein-coupled receptors on the surface of epithelial and immune cells can bind to acetate, propionate, and butyrate, thereby inducing anti-inflammatory cytokine differentiation of Treg cell (Macia et al., 2015). SCFAs promote inflammasome activation by binding to the PYRIN domain of apoptosis-associated speck-like protein, and the activated inflammasomes inhibit *S. typhimurium* through apoptosis (Tsugawa et al., 2020). Moreover, studies have shown that butyrate and propionate upregulate the production of TGF-β1, a cytokine promoting an anti-inflammatory regulatory effect, in intestinal epithelial cells. Butyrate can promote histone acetylation and induce anti-inflammatory responses in Tregs and macrophages (Chang et al., 2014). Furthermore, a study found that butyrate increases the expression of the vimentin protein, which is involved in the killing of bacteria, in infected chicken macrophage cells, thereby inhibiting bacterial invasion of the gut. SCFAs not only promote the production of inflammatory mediators by macrophages but also induce the production of superoxide by neutrophils, which promotes phagocytosis of bacteria by neutrophils (Gupta et al., 2021). As mentioned above, SCFAs can also maintain immune homeostasis by reducing pro-inflammatory responses. For example, butyrate reduces the inflammatory response of macrophages exposed to inflammatory microbial molecules such as lipopolysaccharide by hindering the activity of histone deacetylase (Chang et al., 2014). Similarly, by activating GPR109a (a receptor for butyrate in the colon), butyrate increases the tolerance response of colonic macrophages and dendritic cells, reduces colonic inflammation, and promotes homeostasis (Singh et al., 2014).

Although the immune system is widely affected by SCFAs, this review focuses on the main inhibitory effects of SCFA-induced changes in the immune response to intestinal pathogens. Recent reviews provide a supplementary summary with a more in-depth look at the effects of SCFAs on host physiology (Blaak et al., 2020; van der Hee and Wells, 2021).

## How pathogenic bacteria counteract the effects of short-chain fatty acids

Even though gut-derived SCFAs can inhibit the colonization and growth of some enteric pathogens to some extent, it is clear that enteric pathogens can overcome this disadvantage and successfully colonize the gut, especially with perturbation of the gut microbiota. SCFAs are ubiquitous and abundant metabolites in the gut; therefore, it is not surprising that enteric pathogens have evolved to sense and respond to these effector molecules to coordinate the expression of their virulence traits (Martens et al., 2018).

A recent study reported that in the intestinal lumen of *Salmonella*-infected germ-free mice, *S. typhimurium* overcame the inhibitory effect of propionate by using propionate as a carbon source for anaerobic respiration. This can be explained by *S. typhimurium* using nitrate produced by the host immune response as an alternative electron acceptor to promote anaerobic respiration after inducing inflammation (Shelton et al., 2022). Similarly, previous studies have also found that *S. typhimurium* has a butyrate utilization-related gene, *ydiD*, which can reduce damage to the invasion ability caused by the metabolic utilization of butyrate. When *S. typhimurium* lacks *ydiD*, invasion gene expression is significantly decreased (Bronner et al., 2018). Moreover, *S. typhimurium* can deplete butyrate-producing *Clostridium* in the gut by manipulating its virulence factors, resulting in increased epithelial oxygenation, and promoting the aerobic expansion of *S. typhimurium* in the intestinal lumen (Rivera-Chavez et al., 2016). In addition to metabolically utilizing SCFAs to counteract adverse effects, bacteria are able to sense SCFAs and alter the expression of their own genes to facilitate colonization of the host. *Campylobacter jejuni* sense microbiota-derived butyrate in the intestines of hosts by the BumSR two-component signal transduction system, thus controlling the function of the cognate BumR response regulator and modulating transcription of colonization factors necessary for infection, ultimately promoting infection and diarrheal diseases (Goodman et al., 2020).

In the process of co-evolution with the host, in order to achieve infection, enteric pathogens sense and respond to the newly encountered host environment, continuously adapt to it, and finally achieve niche adaptation and successful colonization (Anderson and Kendall, 2017). Overall, pathogens that expand in the gut under specific circumstances are well-adapted to utilize SCFAs to counteract adverse environments and fuel their expansion.

## Conclusion and outlook

The role of SCFAs, produced by gut microbiota, in maintaining human health has attracted widespread attention. An increasing number of researchers believe that SCFAs are beneficial to gut health because of their anti-pathogenic properties. The mechanism by which SCFAs modulate enteric pathogens is complex, not only involving the direct regulation of pathogenic bacteria but also playing an important role in regulating host physiology and immunity. Endogenous regulatory mechanisms, such as the PTMs of key bacterial proteins, provide potential targets for the control of enteric pathogens. In response to bacterial infection, SCFAs are essential for maintaining gut homeostasis as a link between the microbiota and immune response. However, even if SCFAs promote host-microbiota homeostasis and immune health to some extent, pathogens can adapt to these adverse environments by utilizing or responding to SCFAs, and thus, successfully infect the host and cause disease. Therefore, the utilization of SCFAs can be considered a relatively simple and cost-effective intervention for the control of enteric pathogens, but this also requires consideration of unique influencing factors, such as the immune status of the host and the specificity of pathogens.

**FIGURE 3:**
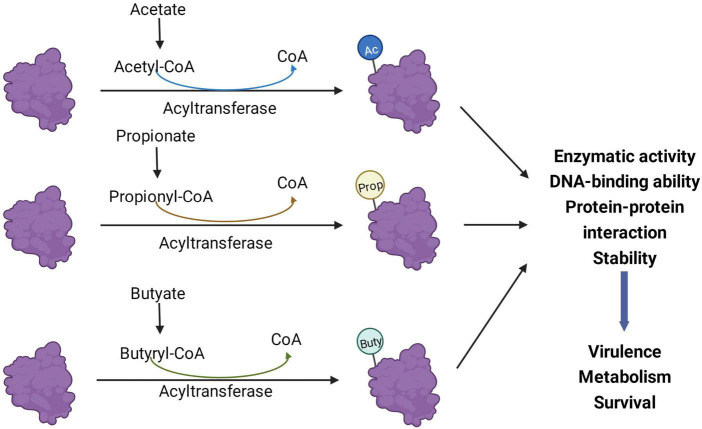
Short-chain fatty acids (SCFAs) regulate bacterial processes through post-translational modifications (PTMs). Acyl-CoA produced by the metabolism of acetate, propionate, and butyrate in bacteria can be different acylation donors for PTMs. After the protein is modified, its enzymatic activity, protein-protein interaction, and DNA binding ability may change, thereby affecting the virulence, metabolism, and survival of bacteria. Ac, acetylation; Prop, propionylation; Buty, butyrylation.

It is a major challenge to determine the exact role of SCFAs in the virulence and growth of pathogenic bacteria and to pinpoint their regulatory mechanisms. Depending on the health status of the human body, SCFAs may vary among individuals and even within different parts of the gut of the same individual. Each SCFA plays a different regulatory role in the intestine, and the composition ratio and concentration of SCFAs greatly affect their regulatory role. Therefore, SCFAs involved in regulating bacterial pathogenicity in the gut may have a complex inter-regulatory relationship between the host and bacteria. With the development and application of new technologies, proteomics and protein modification omics will play a significant role in bacterial research. The effects of SCFAs on intestinal pathogens extend beyond virulence phenotype effects and transcriptional levels. A continually emerging body of evidence shows that SCFAs and their metabolic byproducts greatly influence the PTMs of bacterial proteins and thus affect bacterial virulence, metabolism, and survival (Figure 3), which may be an aspect that needs to be focused on in future research.

Understanding the specific regulatory mechanisms of SCFAs on pathogenic bacteria and the possible response defense mechanisms of pathogenic bacteria may allow SFCAs to be scientifically and rationally utilized to control infections caused by intestinal pathogens. The gut environment is a complex ecosystem, and further research is necessary to fully understand the detailed mechanism by which SCFAs coordinate host defense against pathogens in the gut. Future studies should further reveal the complex relationship between SCFAs and enteric pathogens.

## Author contributions

ZZ drafted and wrote the manuscript. HT and YZ participated in the manuscript correction. XH and MX critically reviewed the manuscript and improved it. All authors gave final approval and agreed to be accountable for all aspects of the work.

## Abbreviations

Acetyl-CoA: Acetyl-coenzyme A
AcP: acetyl phosphate
AIEC: adherent-invasive *E. coli*
EPEC: enteropathogenic *E. coli*
*L. monocytogenes*: *Listeria monocytogenes*
LEE: locus of enterocyte effacement
MRSA: methicillin-resistant *Staphylococcus aureus*
PTMs: post-translational modifications
PPAR-γ: peroxisome proliferator-activated receptor gamma
*S. typhimurium*: *Salmonella typhimurium*
SPI-1: *Salmonella* pathogenicity island-1
SCFAs: short-chain fatty acids
TCA: tricarboxylic acid.
